# Computational expression deconvolution in a complex mammalian organ

**DOI:** 10.1186/1471-2105-7-328

**Published:** 2006-07-03

**Authors:** Min Wang, Stephen R Master, Lewis A Chodosh

**Affiliations:** 1 Departments of Cancer Biology, Medicine, and Cell & Developmental Biology, and the Abramson Family Cancer Research Institute, University of Pennsylvania, 612 BRB II/III, 421 Curie Blvd, Philadelphia, PA 19104, USA; 2 Department of Pathology and Laboratory Medicine, University of Pennsylvania, 613A Stellar-Chance Labs, 422 Curie Blvd., Philadelphia, PA 19104, USA

## Abstract

**Background:**

Microarray expression profiling has been widely used to identify differentially expressed genes in complex cellular systems. However, while such methods can be used to directly infer intracellular regulation within homogeneous cell populations, interpretation of *in vivo *gene expression data derived from complex organs composed of multiple cell types is more problematic. Specifically, observed changes in gene expression may be due either to changes in gene regulation within a given cell type or to changes in the relative abundance of expressing cell types. Consequently, *bona fide *changes in intrinsic gene regulation may be either mimicked or masked by changes in the relative proportion of different cell types. To date, few analytical approaches have addressed this problem.

**Results:**

We have chosen to apply a computational method for deconvoluting gene expression profiles derived from intact tissues by using reference expression data for purified populations of the constituent cell types of the mammary gland. These data were used to estimate changes in the relative proportions of different cell types during murine mammary gland development and Ras-induced mammary tumorigenesis. These computational estimates of changing compartment sizes were then used to enrich lists of differentially expressed genes for transcripts that change as a function of intrinsic intracellular regulation rather than shifts in the relative abundance of expressing cell types. Using this approach, we have demonstrated that adjusting mammary gene expression profiles for changes in three principal compartments – epithelium, white adipose tissue, and brown adipose tissue – is sufficient both to reduce false-positive changes in gene expression due solely to changes in compartment sizes and to reduce false-negative changes by unmasking genuine alterations in gene expression that were otherwise obscured by changes in compartment sizes.

**Conclusion:**

By adjusting gene expression values for changes in the sizes of cell type-specific compartments, this computational deconvolution method has the potential to increase both the sensitivity and specificity of differential gene expression experiments performed on complex tissues. Given the necessity for understanding complex biological processes such as development and carcinogenesis within the context of intact tissues, this approach offers substantial utility and should be broadly applicable to identifying gene expression changes in tissues composed of multiple cell types.

## Background

High-throughput transcriptional profiling using DNA microarrays has enabled routine measurements of genome-wide regulatory changes in a variety of contexts. This technique has been applied to the analysis of gene expression within relatively homogeneous cellular populations as well tissues or tumors consisting of disparate cell types. Within metazoans, cells depend upon environmental signals for growth, differentiation, and survival. However, the interpretation of gene expression profiles obtained in metazoan organisms is complicated by their characteristically complex cellular environments. This results from the fact that microarray expression measurements from heterogeneous tissues with distinct cellular compartments reflect weighted averages of expression levels within different cellular populations. Therefore, observed changes in gene expression may result from *bona fide *changes in regulation within a given cellular compartment, or from changes in the abundance of an expressing compartment within the tissue as a whole. As a consequence, changes in compartment size may be mistaken for the intracellular regulation of gene expression; conversely, genuine regulation within a given cell type may not be detected due to changes in the abundance of cellular compartments that mask its contribution to the tissue as a whole.

To date, several approaches have been used to identify changes in gene expression that occur in different cellular compartments within tissues or tumors comprised of multiple cell types. Laser capture micro-dissection (LCM) [1-3] has been used to physically separate defined cell populations prior to gene expression analysis. A drawback of this approach, however, has been the difficulty in obtaining sufficient quantities of purified material to perform robust, reproducible genome-wide profiling. Other techniques for physical separation may also be used [4,5], however, it is often difficult to ensure that the separation process itself does not introduce substantial alterations in gene expression.

Recently, Lu *et al*. described a computational approach for estimating proportions of cells at specified points in the cell cycle within asynchronous cultures of yeast [6]. Application of this method to complex tissues in higher organisms, however, requires the identification of cell type-specific genes whose expression levels are not substantially affected by biological state or experimental perturbation.

The mammary gland contains two major cellular compartments – epithelial and stromal – that are themselves composed of multiple cell types. These include luminal, myoepithelial, and alveolar epithelial cells, endothelial cells, fibroblasts, white adipocytes, brown adipocytes, and other stromal cell types including multiple hematopoietic cell lineages. During mammary gland development as well as tumorigenesis, the proportions of these compartments change dramatically relative to each other. For example, the epithelial compartments proliferate rapidly and expand during both puberty (ductal elongation) and pregnancy (lobuloalveolar development). Conversely, large scale apoptosis of alveolar epithelial cells and remodeling of the extracellular matrix occurs following pup weaning, thereby returning the gland to a state superficially resembling that of the adult nulliparous animal [7]. In an analogous manner, during the process of tumorigenesis, oncogene activation expands the size of the epithelial compartment while inducing marked changes in other cellular compartments, such as the adipose and fibroblastic stroma and cells of the innate and adaptive immune system.

In this article, we describe and apply a novel extension of a computational deconvolution strategy observed patterns of differentially regulated gene expression. We further demonstrate the utility of this approach by using it to deconvolute expression changes that occur over the course of mammary gland development as well as in response to oncogenic Ras activation within the mammary gland. Predicted gene expression changes computed in this manner were confirmed experimentally and revealed statistically significant regulation of underlying functional pathways that were not detected by conventional gene expression analysis methods.

## Results

### Identification of discriminant gene lists

We hypothesized that changes in the proportions of different cell types within a complex organ could be quantitatively assessed using panels of transcripts that were specifically expressed within each of the composite cellular compartments. In order to identify such transcripts, we selected highly enriched reference samples containing largely homogeneous cell populations and compared gene expression levels in these samples to those in samples representing other cell types. Mammary epithelial cells (MEC), brown adipose tissue (BAT), white adipose tissue (WAT), T cells (CD4+ and CD8+), B cells, plasma cells, macrophages, and fibroblasts were selected for thismodeling approach either because they represent abundant cell populations within the mammary gland or because they are known to play a role in mammary gland development and tumorigenesis.

Microarray expression data were used to generate cell type-specific gene lists through pairwise comparisons of expression between all samples as described in *Methods*. Only genes that showed significant enrichment within a given cell type compared to all other cell types were retained for further analysis. These lists were further refined using stepwise discriminant analysis to identify the optimal cell type-specific transcripts for classification purposes. The resulting gene lists for each cell or tissue type are shown in Table 1. Examination of the genes identified by this discriminant analysis revealed many that had previously been reported to be expressed in a cell type-specific manner. These included genes whose expression is specific for mammary epithelial cells (*Krt2-8*, *Krt1-18*, *Krt1-19*), fibroblasts (*Fgf7*), WAT (*Retn*), BAT (*Ucp1*, *Dci*), plasma cells (immunoglobulin genes), macrophages (*Emr1*), B cells (*CD19*, *CD22*, *CD72*, *Blk*), and CD8+ T-cells (*CD8a*, *CD8b*). These findings support the validity of this approach to identify cell type-specific genes.

### Expression deconvolution during mammary gland development

Deconvoluting expression patterns in a complex tissue using cell type-specific gene lists is dependent on the extent to which the genes selected are expressed at a constant level on a per-cell basis over a range of biological conditions. Moreover, it is also necessary to retain a large enough gene list such that the overall estimate will be robust in the face of biological and technical sources of variation in gene expression measurements [8]. We reasoned that the expression profiles for highly regulated cell type-specific genes would deviate substantially from the average expression profile exhibited by genes within that particular cell type-specific gene list.

**Figure 1:**
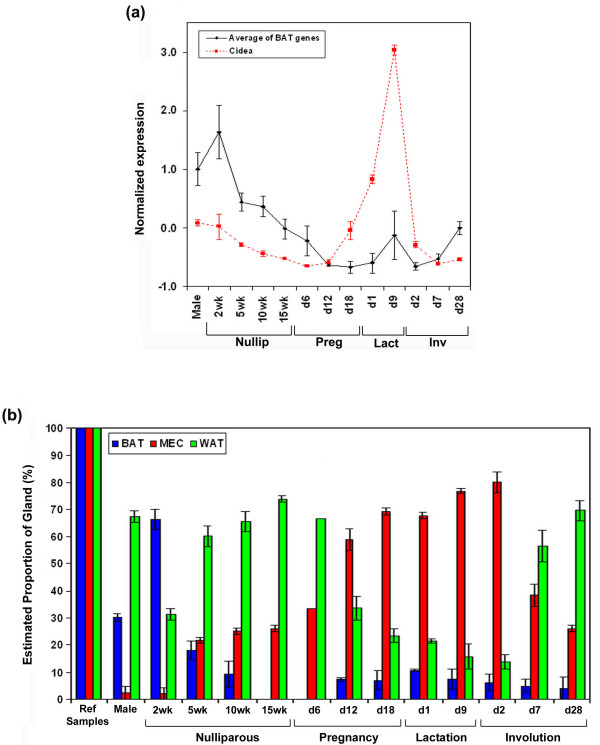
**Expression profiles of BAT-specific genes and estimated changes in cell compartment sizes during mammary gland development**. (a) Normalized expression profile of *Cidea *across mammary gland development stages showing marked deviation during late pregnancy and lactation from the average profile of 10 BAT-specific genes listed in Table 1. Mean expression (±SEM) is shown at each development stage, including nulliparity (Nullip), pregnancy (Preg), lactation (Lact), and involution (Inv). (b) Calculated proportions of brown adipose tissue (BAT), white adipose tissue (WAT), and mammary epithelial cell (MEC) compartments in pure reference samples and mammary tissues at the indicated mammary developmental time points (mean ± SEM).

To exclude highly regulated, cell type-specific genes from consideration, mean and variance-normalized expression profiles across mammary gland development were compared for each cell type-specific gene list. Average or canonical gene expression profiles were calculated for each set of cell type-specific genes. Genes with a Pearson correlation coefficient less than 0.5 when compared to the average group expression profile were excluded from further analysis. For example, BAT-specific genes typically displayed high levels of expression in the mammary glands of male mice and female mice prior to puberty (2 wk G0P0). Expression levels progressively decreased thereafter, reaching a stable nadir during mid-late pregnancy, lactation, and early involution (Figure 1a). This pattern parallels the change in abundance of this cellular compartment during mammary gland development [7]. In contrast, expression of the BAT-specific gene, *Cidea*, was markedly upregulated in the mammary gland during late pregnancy and lactation compared to other BAT-specific genes and was therefore excluded as a marker for BAT in subsequent analysis (Figure 1a). An analogous approach was taken for each list of cell type-specific genes.

To determine whether this method permits the accurate calculation of the relative contribution of different cellular compartments within the mammary gland, we next used these cell type-specific gene lists to estimate compartment sizes across thirteen stages of mammary gland development. We have previously shown that changes in the relative proportions of mammary epithelial cells, WAT, and BAT across mammary gland development substantially affect gene expression profiles observed in the mammary gland [7]. Because these represent the most abundant compartments within the mammary gland, marked changes in their relative sizes during mammary development would be predicted to result in numerous changes in gene expression when expression levels are measuredwithin the tissue as a whole.

Expression levels for cell type-specific genes representing MEC, BAT, and WAT were averaged across the three reference samples for each cell type. The resulting values for each gene were taken as its basal expression within its cognate tissue compartment. Mean and variance-normalization was first performed across all genes in that sample, and proportions of each cell type were estimated by obtaining solutions to linear equations of the form **Ax **= **y**, where **A **is an *m *× *n *matrix of expression values (*m *genes × *n *reference groups), **y **is a vector of *m *values in the test sample, and **x **is the vector of *n *values reflecting the estimated relative proportions of each cell type within the mixture. Solutions were estimated using simulated annealing [6]. A related approach, albeit using different methods for identifying genes to be used in the estimate, has been described by Lu *et al*. who termed the process of estimating cellular proportions "expression deconvolution" [6].

**Figure 2:**
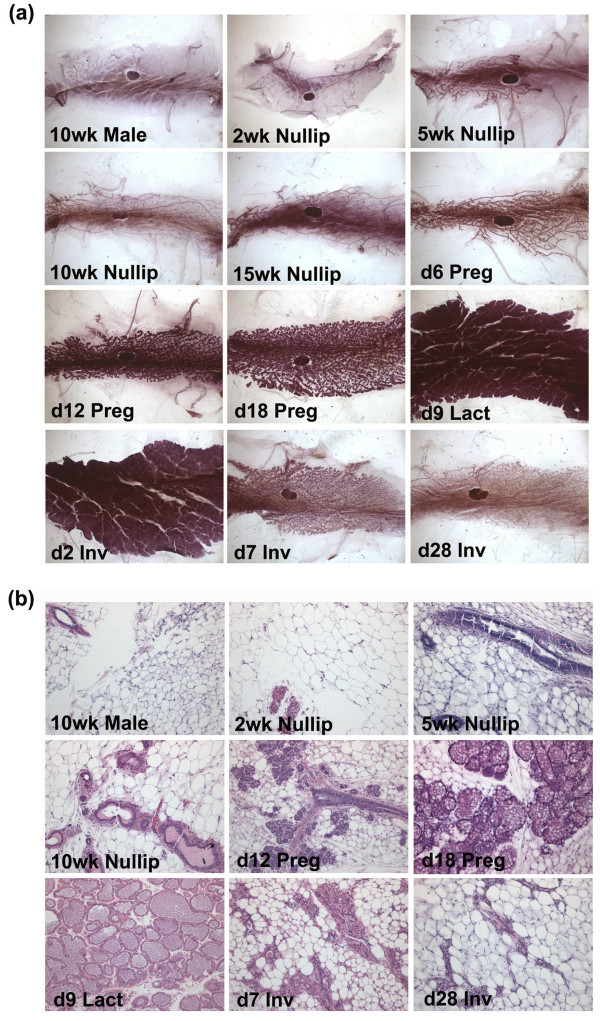
**Mammary gland morphology during the development**. (a) Analysis of carmine-stained whole mounts (magnification 6×) and (b) hemotoxylin and eosin (H&E)-stained sections (magnification 100×) showing the relative changes in size of the epithelial and adipose compartments of the number 4 mammary gland at the indicated time points during mammary gland development.

Expression deconvolution was first applied to the initial expression data sets derived from the MEC, WAT, and BAT purified cell populations. As expected, this algorithm correctly estimated the composition of each of the nine samples as consisting entirely of its appropriate corresponding cell type (Figure 1b). Next, expression deconvolution was performed on data sets representing triplicate measurements of 13 time points spanning mammary gland development (Figure 1b). This analysis yielded estimates of the proportions of MEC, WAT, and BAT present in the mammary gland during each stage of development. These estimates closely paralleled changes in compartment sizes that were observed by histological analysis, thereby confirming the validity of this approach (Figure 2a and 2b). For example, in male and 2-week-old female mice, only a rudimentary epithelial tree is present in the mammary fat pad (Figure 2a and 2b). Consistent with this, expression deconvolution analysis estimated that the mammary gland at these stages is composed primarily of brown and white adipose tissue, with only a small amount (<5%) of epithelium (Figure 1b). Also consistent with previous descriptions, the estimated proportion of BAT declines during puberty to reach a nadir in adult nulliparous mice (Figure 1b and [7]). Conversely, during this same period, ductal elongation and branching occur with extension of mammary epithelial ducts into the surrounding fat pad (Figure 2a and 2b). This process results in a substantial increase in the proportion of epithelial cells in the mammary gland between 2 wk and 10 wk of age, at which time ductal morphogenesis is largely complete. This increase in the epithelial content of the gland is accurately reflected in cell compartment estimates using expression deconvolution (Figure 1b).

Similar to the expansion of the epithelial compartment during puberty, further increases in epithelial content occur during pregnancy due to the expansion of the alveolar epithelial compartment (Figure 2a and 2b) and these changes are also accurately captured by expression deconvolution (Figure 1b). Most of the observed increase in epithelial content occurs by d12 of pregnancy, consistent with the decline in epithelial proliferation rates after this stage of lobuloalveolar development (Figure 1b and [9]). As reflected both by morphology and expression deconvolution estimates, the proportion of epithelial cells peaks during late pregnancy and lactation (Figure 1b, 2a, and 2b). Finally, following the weaning of pups, programmed cell death and remodeling of the maternal gland during postlactational involution result in a decrease in the size of the epithelial compartment and a corresponding increase in the relative size of the WAT compartment (Figure 1b, 2a, and 2b). Thus, in aggregate, the calculated proportional composition of the mammary gland with respect to the contribution of each of these three cellular compartments across mammary development is consistent with previously described changes as well as direct visualization of these compartments in staged samples.

### Effects of compartment size adjustment on the identification of regulated genes

A principal benefit of the ability to accurately estimate changes in compartment sizes is the possibility of distinguishing *bona fide *changes in gene expression within a compartment from apparent gene expression changes due solely to changes in compartment size. That is, since the overall expression level in the mammary gland for a gene whose expression is not regulated is equivalent to the sum of its expression levels within each cellular compartment, it should be possible to quantitatively predict the changes in that gene's apparent expression level that would result solely from specified changes in the abundance of cellular compartments within the gland.

**Figure 3:**
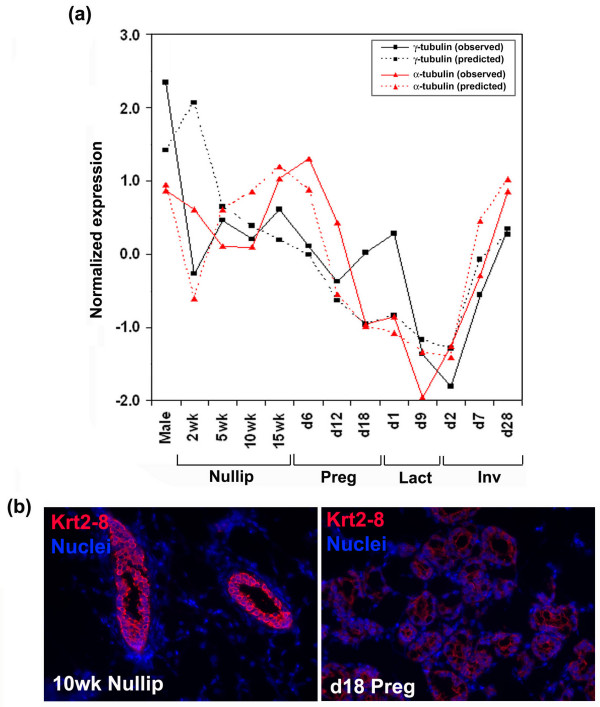
**Tubulin and cytokeratin 8 expression during mammary gland development**. (a) Predicted (dashed lines) and observed (solid lines) normalized expression for α-tubulin and γ-tubulin during mammary gland development. A high correlation was found between observed expression profiles and predicted profiles calculated based on changes in cell compartment sizes. (b) Immunofluorescence staining of cytokeratin 8 (Krt2-8) showing downregulated expression in the mammary epithelium at d18 of pregnancy compared to 10 wk of nulliparous develoment.

To test the validity of this approach, we calculated expression levels for α- and γ-tubulin across mammary development based on their expression within each compartment within the gland. We then compared these predicted expression levels to those observed across development. Briefly, the average expression levels observed for the tubulin genes within the reference MEC, WAT, and BAT samples were used in conjunction with the estimated proportions of the three compartments at each of the development stages to generate a predicted expression values for the tubulin genes (Figure 3a). The resulting calculated expression levels were largely congruent with the observed values with correlation coefficients greater than 0.6.

To ascertain whether this expression deconvolution method could identify intrinsic gene expression changes, mammary glands harvested from late pregnant (day 18) mice were compared to those from 10-week-old nulliparous females. These two developmental stages have marked differences in physiology as well as in the abundance of epithelial and adipocyte compartments [10] and thereby provide an appropriate test for the adequacy of this approach. First, the predicted expression level for each gene was calculated based on its expression level in the MEC, WAT, and BAT compartments, coupled with changes in the sizes of the three compartments between these two stages of mammary gland development. This predicted gene expression level was then subtracted from the observed expression level, and the resulting values for these two developmental points were compared using the Statistical Analysis for Microarrays (SAM) algorithm [11] with a false discovery rate threshold (FDR) of <3%. Additional File 2 lists the results of SAM comparisons both before and after taking into account changes in compartment sizes and Table 2 summarizes the number of probesets whose regulation calls were altered. Table 3 lists selected genes that were considered to be differentially expressed either before or after adjusting for changes in cell compartment sizes.

The effects of taking compartment size changes into account was substantial as 20.2%, 35.2%, and 15.5% of up-, down-, and non-changing calls, respectively, were altered as a consequence of adjusting for changes in cell compartment sizes (Table 2) that occur during mammary development. As shown in Table 2, 16% of genes called upregulated prior to signal adjustment were called non-changing and 4.2% were called downregulated, after taking into account changes in cell compartment sizes. Thus, adjustment for cell compartment size alters change calls for 20.2% of genes initially called upregulated. Similarly, 35.2% of genes initially called downregulated were predicted to be either up-regulated (3%) or non-changing (32.2%) after taking into account changes in cell compartment sizes. These findings strongly suggest that apparent changes in expression for a substantial fraction of genes identified as differentially regulated using standard analytical approaches may actually reflect changes in cell compartment sizes that occur during mammary gland development rather than intrinsic gene regulation.

For example, examination of genes in Table 3 revealed that a number of adipocyte-specific genes, such as *Pparg *and *Fabp4 *(*aP2*), appeared to be significantly downregulated in the pregnant (d18) compared to the 10-week-old nulliparous gland prior to adjustment for estimated changes in cell compartment sizes. After adjusting for estimated changes in cell compartment sizes, however, these adipocyte-specific genes were no longer considered to change significantly between these two developmental time points. This suggests that the apparent down-regulation of multiple WAT-specific genes that occur during pregnancy is most likely a consequence of a decrease in the size of the adipose compartment that occurs at this stage (see Figure 1b, 2a, and 2b).

Figure 1b implies that the contribution of the adipocyte compartment to total mammary gland mRNA at d18 of pregnancy is less than half that of its contribution to the 10 wk nulliparous gland. As such, the apparent expression of genes that are expressed predominantly within the WAT compartment would be predicted to decrease from 10 wk nulliparous development to d18 of pregnancy. Consistent with this, the pre-adjustment level of leptin (*Lep*) expression appeared significantly downregulated from 10 wk nulliparous gland to d18 of pregnancy, whereas post-adjustment signals indicated that its expression level did not differ significantly between these two developmental stages. In fact, we have previously shown by *in situ *hybridization that *Lep *mRNA expression does not change substantially on a per-cell basis between puberty and early pregnancy [7], providing experimental confirmation of this computational result.

Conversely, a substantial fraction of genes (15.5%) that were not considered to be differentially regulated prior to adjustment for changes in cell compartment sizes were found to be either up- (5.2%) or downregulated (10.3%) following deconvolution (Table 2 and 3). This suggests that changes in cell compartment sizes that occur during mammary gland development mask *bona fide *changes in expression for a substantial number of genes. For example, lipoprotein lipase (*Lpl*), insulin-like growth factor 2 (*Igf2*), and prolactin-like protein E (*Prlpe*) were all identified as upregulated in late pregnancy compared with the 10 wk nulliparous gland only after taking into account the changes in cell compartment sizes. This finding is consistent with previous reports that the content and activity of *Lpl*, which is expressed in mammary adipocytes, increases within the fat pad during pregnancy [12] and early lactation [13]. Similarly, the adipocyte-secreted factors *Igf2 *and *Prlpe *were found to be upregulated only after adjusting for the decreasing sizes of the adipocyte compartment that occurs during pregnancy. This finding is consistent with their previously described role in promoting mammary gland proliferation and differentiation [14,15].

Several genes encoding transporter proteins (e.g., *Abca1*, *Abcd2*, *Abcc9*) appeared to be down-regulated in pregnancy prior to adjusting for changes in compartment sizes, but were predicted to be up-regulated following signal adjustment; these results are consistent with the preparation of the mammary gland for large-scale transport and secretion during lactation. Conversely, pro-apoptotic genes such as *Bad*, *Bax*, *Bid*, and *mdm2 *were found to be downregulated at d18 of pregnancy, consistent with the low levels of apoptosis observed during late pregnancy and lactation, only after adjusting for changes in compartment sizes (Table 3). As such, the congruence of these results with our current understanding of mammary gland physiology suggests that this deconvolution approach may be useful for identifying intrinsically regulated genes within heterogeneous tissues.

Notably, the expression of several epithelial genes, including *Krt2-8*, *Krt1-18*, and *Krt1-19*, did not appear to change between 10 wk nulliparous and d18 pregnant mice prior to adjusting for changes in compartment sizes, but were predicted to be significantly downregulated after taking the compartment size changes account. These results were surprising not only because developmental regulation of these epithelial markers within the mammary gland has not previously been reported, but also because two of these cytokeratin genes (*Krt2-8 *and *Krt1-18*) were themselves members of the epithelial-specific set of transcripts used to adjust gene expression for compartment size. Closer examination of these data revealed that a modest increase in apparent *Krt2-8 *and *Krt1-18 *expression levels in late pregnancy is offset by more substantial upregulation of other epithelium-specific genes, such as *Ndrg1*. To determine whether this prediction was accurate, Krt2-8 protein expression was assessed by immunofluorescence (Figure 3b). This analysis suggested that Krt2-8 expression does indeed decrease on a per-cell basis within the murine mammary epithelium during late pregnancy. This, in turn, indicates that adjusted expression values can accurately identify differentially regulated genes whose unadjusted expression values within the mammary gland as a whole do not appear to change.

### Effect of compartment size adjustment on GO analysis

To identify coherent changes in functional gene expression patterns between late-pregnant (d18) and 10 wk nulliparous mice, statistically significant associations between Gene Ontology (GO) categories and lists of up- and down-regulated genes were identified using EASE before and after adjusting for estimated changes in cell compartment sizes [16]. Table 4 lists a subset of the significant associated terms in the Biological Process category and Additional File 3 lists the significant GO terms. This analysis revealed that GO terms relating to protein localization and protein transportation were significantly associated with up-regulated gene lists irrespective of adjustment for changes in cell compartment sizes. In contrast, while pre-adjustment gene lists suggested the down-regulation of multiple metabolic pathways (fatty acid metabolism, lipid metabolism, and carboxylic acid metabolism), these associations were absent in the post-adjustment gene list. This indicates that the initial associations observed were most likely a result of the diminished size of the WAT compartment during late pregnancy rather than *bona fide *regulation within an expressing compartment.

### Deconvolution of oncogenic Ras activation

Having demonstrated the ability of expression deconvolution toidentify gene expression changes in the setting of marked changes inthe proportions of different cellular compartments, we wished to use this approach to examine gene expression changes that occur in the mammary gland as a consequence of oncogene Ras activation. Our laboratory has previously described the mammary-specific, doxycycline-inducible expression of oncogenes using bitransgenic mouse models [17-20]. For the present experiments, an activated *Ras *transgene was placed under the control of a tetracycline-dependent minimal promoter, and Ras was induced in the mammary gland for specified periods of time by administering animals 2 mg/ml doxycycline in their drinking water. Mammary tissues were harvested from nine mice each at day 0 (d0), day 1 (d1), day 2 (d2), day 4 (d4), day 8 (d8), and day 14 (d14) of doxycycline treatment and oncogene induction. RNA prepared from these samples was used to generate three independent pooled samples, each consisting of RNA from three animals, and microarray transcriptional profiling was performed on Affymetrix MG-U74Av2 arrays.

**Figure 4:**
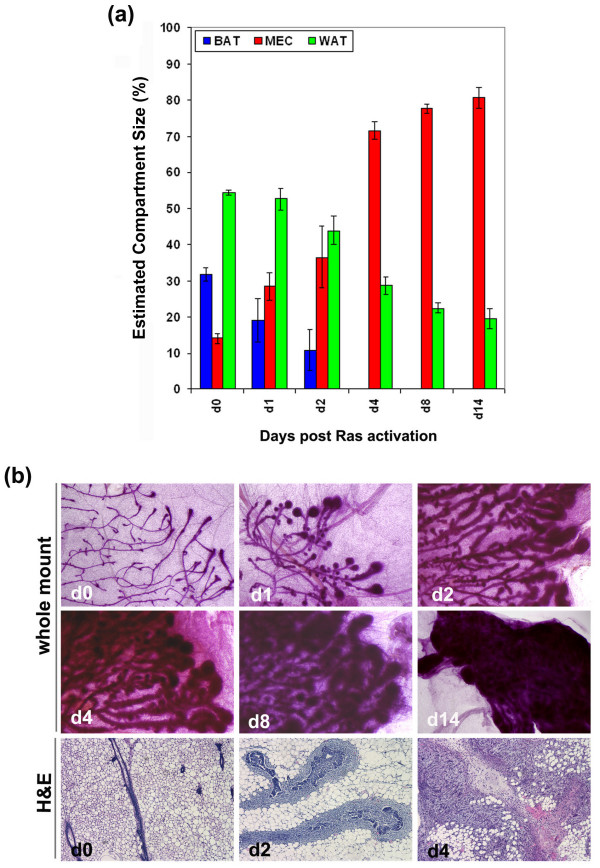
**Changes in cell compartment sizes following Ras activation in the mammary gland**. (a) Calculated proportions of BAT, WAT, and MEC compartments in the mammary gland at the indicated days following oncogenic Ras activation in MTB/TRAS mice (mean ± SEM). Ras activation expands the epithelial compartment and decreases the adipocyte compartment. (b) Carmine-stained whole mounts (magnification 10×) and H&E-stained sections (magnification 100×) of number 3 mammary glands in MTB/TRAS mice showing changes in the sizes of cell compartments following oncogenic Ras activation similar to those predicted by deconvolution analysis.

As was performed for mammary development, tissue-specific gene lists from epithelial cells, WAT, and BAT were first used to estimate changes in the relative proportions of these three compartments following Ras induction (Figure 4a). Expression deconvolution predicted that Ras induction resulted in the dramatic expansion of the epithelial compartment with a corresponding loss of BAT and WAT (Figure 4a). Aanalysis of mammary whole mounts and hematoxylin and eosin (H&E)-stained sections confirmed that after 4 days of Ras induction, the mammary epithelial compartment occupied more than 70% of the mammary gland (Figure 4b). As was the case for normal mammary gland development, calculated estimates of changes in cell compartment sizes derived from gene expression analysis (Figure 4a) closely paralleled morphological changes observed in the mammary gland induced following Rasactivation (Figure 4b).

We next adjusted gene expression levels to account for the calculated baseline expression that would be attributable to the estimated proportions of the three major cellular compartments (MEC, WAT, and BAT). Gene expression data were then re-analyzed to identify genes that were differentially regulated following 4 days of Ras activation. Additional File 4 lists gene expression change calls before and after the adjusting for estimated changes in cell compartment sizes. Table 2 summarizes the number of probesets with altered change calls, and Table 5 lists selected genes that were considered to be differentially expressed either before or after adjusting for changes in cell compartment sizes.

As observed for analyses of mammary development, adjustment for changes in cell compartment sizes identified genes whose apparent expression changes actually reflected changes in the sizes of their expressing cell compartment, as well as genes whose changes in expression between these two points had been masked by changes in cell compartment sizes. These effects were substantial as 32.4%, 58.3%, and 18.9% of up-, down-, and non-changing calls, respectively, were altered as a consequence of adjusting for changes in cell compartment sizes (Table 2).

**Figure 5:**
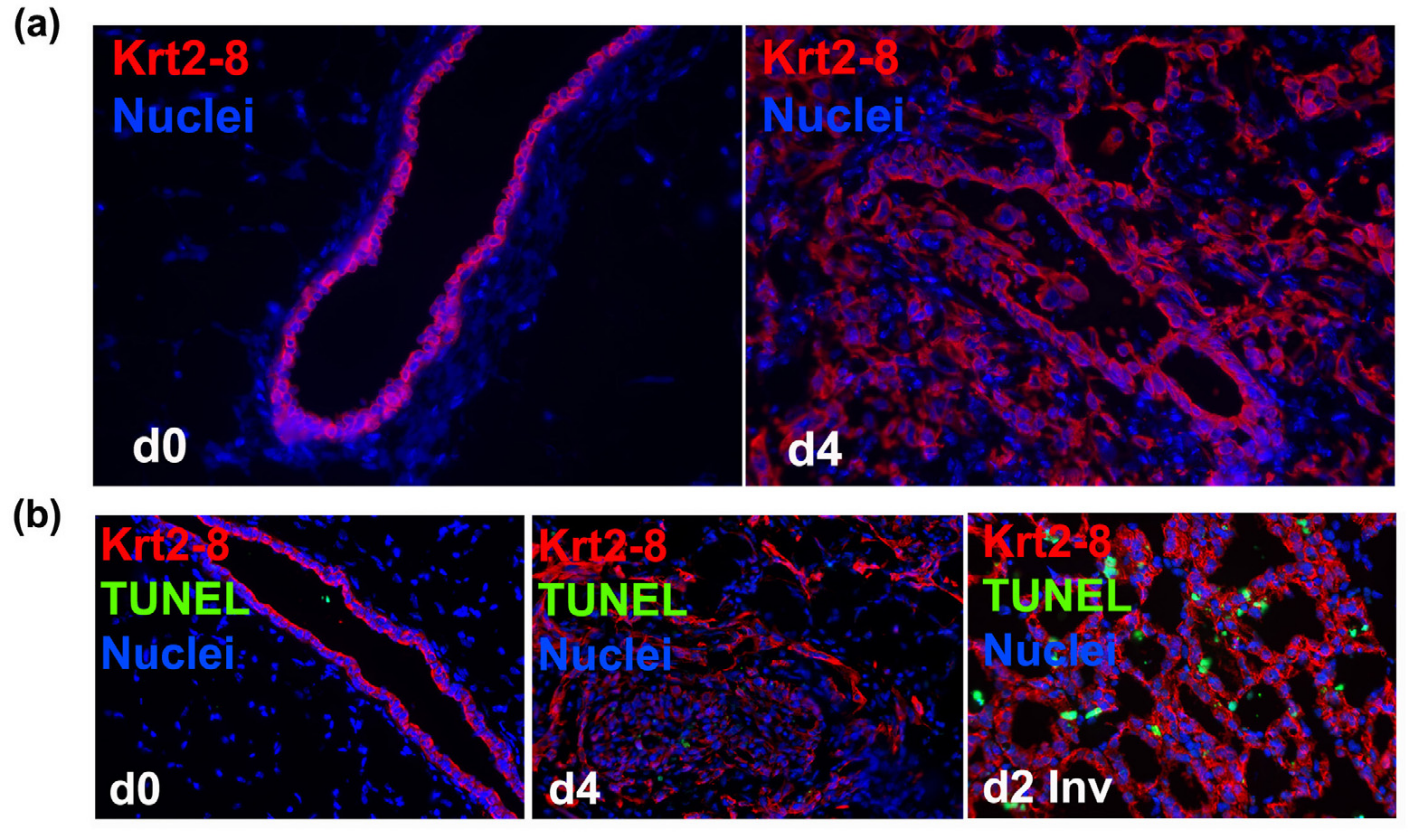
**Cytokeratin 8 expression and apoptosis in the mammary gland following Ras activation**. (a) Immunofluorescence staining for cytokeratin 8 (Krt2-8) in the mammary gland at d0 and d4 following oncogenic Ras activation revealed no significant changes in its expression within epithelial cells. (b) TUNEL analysis showing that Ras activation does not increase apoptosis rates in the mammary glands following four days of Ras activation.

As was observed for the analysis of pregnancy-induced changes in gene expression in the mammary gland, the expression of adipocyte-specific genes such as *Dci*, *Ucp3*, *Cepba*, *Lep*, *Pparg*, *Fsp27*, and *Fabp4 *that appeared to be down-regulated prior to adjustment for cellular compartment sizes, were no longer considered to be changed after adjustment. This suggests that the apparent down-regulation of these genes was due not to Ras-induced repression of gene expression, but to the reduction in size of the adipocyte compartment that occurs as a consequence of the epithelial expansion triggered by Ras activation. Similarly, although *Krt2-8 *and *Krt1-18 *appeared upregulated in response to Ras activation prior to adjustment, these genes were not considered to be differentially expressed after deconvolution. This suggests that the increase in cytokeratin expression observed following 4 days of Ras activation principally reflects expansion of the epithelial compartment rather than intrinsic regulation of these genes within the epithelial compartment. Consistent with the deconvolution analysis, significant changes in per-cell expression of cytokeratin 8 protein levels were not observed following immunofluorescence analysis performed at these two time points (Figure 5a). Similar to cytokeratins, the pro-apoptotic genes *Bax*, *Bid*, and *Usp3 *no longer showed significant up-regulation after adjusting for expansion of the epithelial compartment. Consistent with this result, TUNEL staining revealed low levels of apoptosis that did not increase following Ras activation (Figure 5b).

As was the case for our analysis of mammary gland development, multiple genes were identified that were predicted to be differentially expressed only after adjusting for changes in cell compartment sizes. Genes that were predicted to be up-regulated following expression deconvolution included *Rras *and *Ctsb*, whereas *Mr1 *was predicted to be down-regulated following adjustment. These predictions are consistent with previously published reports on the effect of Ras activation [21,22], providing additional evidence that this approach can reliably adjust expression profiles for changes in compartment sizes.

Having adjusted Ras-induced changes in gene expression levels for changes in cell compartment sizes, we next analyzed lists of differentially regulated genes for statistical association with Gene Ontology annotation. Analysis of pre-adjusted gene lists revealed significant associations between down-regulated genes and multiple energy-related pathways, including glycolysis, lipid metabolism, carbohydrate metabolism, fatty acid metabolism, and electron transport (Additional File 5 and Table 6). This result was surprising given the known ability of Ras to stimulate glycolysis [23] as well as the presumably large energy requirement for macromolecular synthesis necessary to sustain oncogene-induced epithelial proliferation. However, after adjusting for the decreased proportion of adipocytes in the mammary gland, these metabolic pathways were no longer significantly associated with down-regulated genes (Table 6). This suggests that the observed pre-adjustment association was principally a consequence of the reduction in size of the WAT compartment induced by Ras activation.

Finally, adjusting for changes in cell compartment sizes revealed several significant associations between up-regulated genes and GO categories that were not evident prior to adjustment. These included genes associated with the inflammatory response and integrin-mediated pathways (Table 6). Ras-mediated activation of these pathways has previously been described [24].

### Comprehensive compartment dynamics in the mammary gland

**Figure 6:**
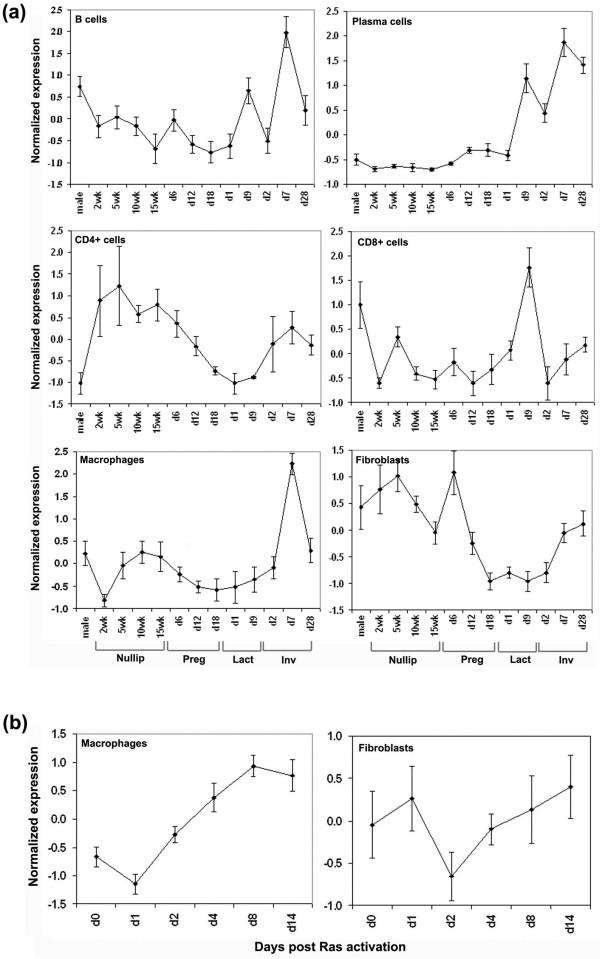
**Estimated changes in the relative abundance of stromal cell types**. Calculated relative abundance (mean ± SEM) of stromal cell types (a) during mammary gland development or (b) following oncogenic Ras activation. Expression levels shown are the average of normalized expression levels of the tissue-specific genes for each tissue/cell type listed in Table 1.

The above findings demonstrate that adjusting mammary gene expression profiles for changes in the size of three principal cellular compartments (MEC, WAT, and BAT) is sufficient both to eliminate false-positive changes in gene expression due to changes in cell compartment sizes and to reveal intrinsic changes in gene expression that are masked by changes of compartment sizes. Consequently, we wished to extend this method for estimating compartment size to include additional cell types for which expression profiling data were available from purified cell populations. These included macrophages, fibroblasts, B-cells, T-cells, and plasma cells. Estimates of relative changes in the size of each cell type compartment were derived as before for mammary gland development and inducible Ras activation (Figure 6).

As shown in Figure 6a, the calculated proportions of hematopoietic cells, including B cells, plasma cells, CD4+ and CD8+ T cells, and macrophages, were found to increase during lactation or early postlactational involution. These findings are consistent with previous reports and with the putative roles of these cell types in antibody secretion into milk and the detection and clearance of apoptotic alveolar debris during postlactational involution [24,25]. Additional increases in macrophage and CD8+ T cell abundance were predicted at the onset of puberty, and fibroblast abundance was estimated to decrease during pregnancy and subsequently increase during involution.

Estimated changes in macrophage and fibroblast cell populations in the mammary gland following Ras activation are shown in Figure 6b. The calculated increase in macrophages following Ras activation suggested macrophage infiltration into the mammary gland. The accuracy of this prediction was supported both by enzyme-linked immunosorbent assay (ELISA) of *IL-1β*, a known potent mediator of immune and inflammatory responses [26], and macrophage infiltration assays at d4 following Ras induction (manuscript in preparation).

**Figure 7:**
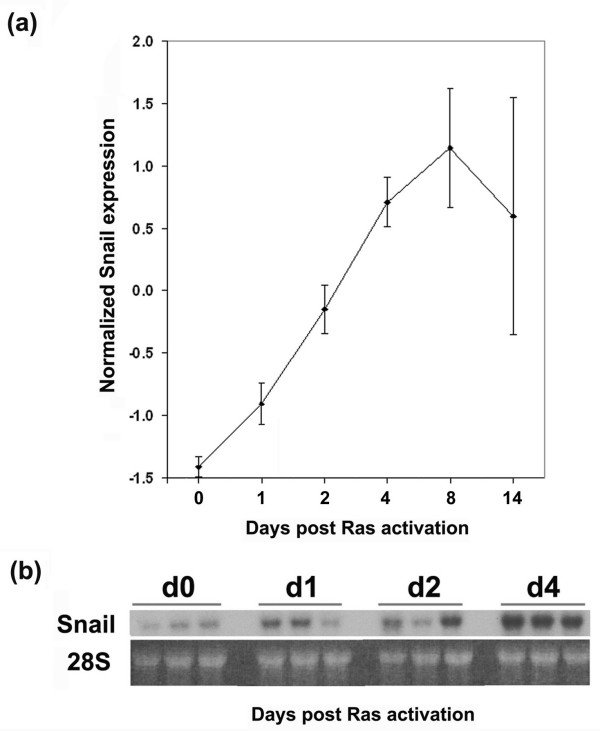
**Upregulation of *Snail *by Ras activation**. (a) Normalized microarray expression profiling (mean ± SEM) or (b) Northern hybridization analysis of *Snail *expression as a function of oncogenic Ras activation.

Notably, while the mammary epithelial compartment constitutes the majority of the mammary gland after 2 days of Ras activation (Figure 4a and 4b), a progressive increase in the estimated proportion of fibroblasts was noted at d4, d8, and d14 (Figure 6b). We considered the possibility that the apparent increase in fibroblast-associated genes might reflect an epithelial-mesenchymal transition (EMT) resulting from Ras activation. To explore this possibility, we examined this data set for the expression of *Snail*, a potent activator of EMT [27,28]. Consistent with our prediction, both microarray gene expression data as well as Northern hybridization analysis showed dramatic up-regulation of *Snail *in response to Ras activation (Figure 7).

## Discussion

While microarray expression profiling can be used to directly infer intracellular gene regulation within homogeneous cell populations, the complex mixture of cell types within tissues of higher organisms substantially hampers the interpretation of results from such experiments. Specifically, changes in gene expression observed within complex tissues may be due either to changes in gene regulation within a given cell type or to changes in the relative abundance of expressing cell types. Given the necessity of understanding complex biological processes such as development and carcinogenesis within the context of intact tissues, this problem is of substantial importance.

Previous attempts to address this problem have focused on purification or enrichment of defined cell types by physical methods, such as laser-capture microdissection or magnetic bead separation [1,2]. While these approaches have been successful, they are labor-intensive and become prohibitive when attempting to analyze large sample collections. As an alternative, we have chosen to apply a computational method for deconvoluting expression profiles derived from intact tissues by using reference data derived from purified populations of the constituent cell types. An analogous approach has previously been used by Lu *et al*. for analyzing cell populations during the yeast cell cycle [6]. However, the more challenging goal of deconvoluting gene expression within a complex metazoan tissue has not previously been reported.

As Lu *et al*. noted in their studies in yeast, a critical element of the deconvolution approach is the choice of purified cell populations that will adequately represent the repertoire of cell types present within the mixed population [6]. In the case of the mammary gland, identification and accurate representation of the multiple cellular compartments present is nontrivial. To attempt to model the major cell types in the murine mammary gland, we chose populations representing mammary epithelial cells, white adipocytes, brown adipocytes, fibroblasts, plasma cells, B cells, T cells (CD4+ and CD8+), and macrophages. Despite the relatively large number of cell types modeled, it is worth noting that even this represents an over-simplification since these cell types may be further subdivided based on lineage (luminal vs. alveolar vs. myoepithelial cells) or differentiation status (preadipocytes vs. mature adipocytes). Moreover, additional cellular compartments, such as those responsible for the mammary vasculature, were not included in the model. It is notable, therefore, that we were able to achieve robust results simply by restricting the deconvolution model to three major cellular components (MEC, WAT, and BAT). This suggests that fluctuations in the sizes of these three compartments account for a significant amount of the variation in gene expression observed during mammary gland development and carcinogenesis.

After identifying the relevant constituent cell types within a tissue, it is then necessary to identify robust sets of reference genes whose expression is specific to a given cell type and relatively unaffected by different physiological and experimental conditions. Cell type-specific genes have previously been identified using a variety of methods, including signal-to-noise calculations [29], linear discriminant analysis [30], and feature relevance approaches [31]. Additionally, a number of epithelial cell type-specific genes within the mammary gland have previously been identified using affinity-purified populations from normal and neoplastic mammary tissues [4]. Many of these mammary epithelial markers, including *Krt2-8*, *Krt1-18*, *Krt1-19*, and *Stard10*, were also identified in the current analysis. We now extend previous work by using these cell type-specific genes to provide quantitative estimates of compartment size and to adjust expression values by taking into account changes in compartment sizes. In the analysis presented here, we have combined a signal-to-noise approach (through T statistic rankings) with linear discriminant analysis in order to identify genes that are preferentially expressed within individual cell types. Although cell type classification cannot be considered equivalent to the problem of estimating the proportional contributions of those cell types to a mixed population, we reasoned that the most discriminating genes for classification were also likely to provide a set of genes from which successful estimates of cell compartment size could be derived.

To attempt to identify genes that would provide useful markers for cell type abundance by identifying those with consistent expression under a variety of biological conditions, we analyzed the behavior of candidate genes in data sets spanning mammary gland development. Since mammary development encompasses a diverse group of biological processes including branching morphogenesis, alveolar differentiation, apoptosis, and extracellular matrix remodeling [10], it provides a useful test set for further gene selection. To identify outlier genes that were significantly regulated during development, we eliminated genes whose expression was not sufficiently correlated with the normalized mean profile of discriminating genes for specific cellular compartments. The fact that this overall approach selected a number of known marker genes specific for each tissue, including *Krt2-8 *and *Krt1-18 *for mammary epithelial cells, *Ucp1 *for BAT, *Retn *for BAT, and *CD8a *and *CD8b *for CD8+ cells, suggests that it was successful. Furthermore, the identification and elimination of genes (such as *Cidea*) with expression patterns that clearly differ from these known marker genes underscores the importance of this step. Optimal extension of this method to other organs and organisms will, in each case, require the collection of data from reference populations under a sufficiently diverse set of biological conditions.

The refined list of co-regulated marker genes and their reference expression values were then used to estimate the proportional contribution of each cell type to the mixture of mammary cell populations by using simulated annealing to estimate solutions to the corresponding linear equations. When this technique was applied to solve equations for the relative contributions of three major mammary cell types across mammary gland development, we achieved quantitative predictions of cellular compartment size changes that were strongly correlated with observed morphological changes. To further confirm the utility of this computational approach, we applied this method to a conditional transgenic mouse model in which oncogenic Ras was inducibly activated in the mammary epithelium. As before, estimated changes in compartment sizes derived from this model were consistent with those observed by morphological analysis of mammary whole mounts and stained sections.

Finally, we extended this expression deconvolution approach to include additional cell types, particularly those of the hematopoietic system. This approach predicted increases in several types of immune cells during lactation and postlactational involution, consistent with previous reports of their role in clearing apoptotic debris and with their persistent presence in the mammary gland following the cycle of pregnancy, lactation, and postlactational involution [24,25]. Interestingly, expression deconvolution also predicted an increase in macrophages and CD8+ cells at the onset of puberty. Although macrophages have previously been shown to be required for ductal elongation and to be recruited to puberty-specific structures termed terminal end buds [32], to our knowledge there has been no similar role described for T cells. Taken together, our results support the contention that the method described can reliably estimate dynamic changes in cellular compartments within a complex mammalian organ.

### Deconvolution-adjusted expression analysis

While the ability to estimate compartment dynamics is useful in its own right, we were particularly interested in attempting to adjust gene expression values derived from whole-organ profiling in order to eliminate apparent changes in expression due solely to compartment dynamics. Furthermore, changes in expression due to changes in compartment sizes can offset genuine alterations in gene expression within particular cell types such that net expression may appear unchanged. Thus, adjusting expression values has the potential to reduce false-positive and false-negatives gene expression changes and to thereby increase both the sensitivity and specificity of differential gene expression experiments.

To adjust the expression signal associated with any given gene on the array, we utilized our estimates of compartment size in conjunction with mean expression values for that gene in each of the reference populations representing different cell types. This yielded a gene expression value that would be expected if its expression in other samples was solely determined by the composition of the sample with respect to each compartment in the absence of regulation within a compartment. The "predicted" value based on compartment sizes was then subtracted from the observed value and SAM was used to identify differentially expressed genes. Assuming that the expression value of a given gene in the reference samples provides a reasonable estimate of basal expression of that gene in the tissue, this comparison of "deconvolution-adjusted" expression values should reduce the number of genes identified as differentially expressed as a consequence of changes in compartment sizes.

When this approach was applied to the analysis of gene expression changes that occur during pregnancy and in response to Ras activation, the post-adjusted change calls of most genes were consistent with previous reports in the literature. For example, *Abcg1*, *Abcg2*, *Csna*, *Csnb*, and *HIF1a *have been previously shown to be upregulated during pregnancy [33], and *Myc *[34], *Ccnd1*, *Cxcl1*, *Tgfb1*, and *Itgb2 *are known to be induced by Ras activation [24]. The current approach retains the upregulated change calls of these genes.

Using this approach, we were also able to identify genes that appeared to be differentially expressed before- but not after-adjusting for changes in cell compartment sizes. These likely correspond to genes whose apparent expression levels change only as a consequence of compartment dynamics. Conversely, we were also able to identify genes whose expression appeared unchanged prior to adjustment, but which were found to be differentially expressed once changes in cell compartment sizes had been taken into account. These may represent genes whose *bona fide *regulation is masked by offsetting changes in compartment sizes. Finally, statistical association of post-adjustment gene expression lists with Gene Ontology (GO) revealed some biological processes that were masked by changes in compartment sizes. Several of the pathways significantly associated with post-adjustment gene expression changes have either been reported in the literature or confirmed experimentally by ourselves, such as the activation of inflammatory response and integrin-mediated pathways induced by Ras activation.

Despite the successful application of this approach to the adjustment of gene expression profiles, several caveats must be considered. First, any given adjusted gene expression profile is critically dependent upon the behavior of that particular gene in the relatively homogeneous and purified cell populations used as the reference samples. That is, adjustments are based on the assumption that "basal" gene expression can be estimated by the average gene expression value in the reference samples from the same organism that are of a given cell type. Thus, if the typical expression levels for a given gene in its relevant compartment *in vivo *is substantially higher than that measured in the purified population, a change due solely to changes in the size of that compartment will be under-compensated and expression of the gene may still appear to change. Thus, while we expect that this method will enrich for genes that are intrinsically regulated within cells, a certain number of "false positive" changes will undoubtedly remain.

Ultimately, the success of this enrichment will depend upon the purified populations used to provide the basis for subsequent estimates. In this regard, we anticipate that our results would be improved by deriving reference expression values from purified primary cell populations derived directly from the mammary gland. Although LCM-based analysis of large numbers of specimens may be difficult, a number of groups have successfully utilized this strategy to purify tumor populations [35,36]. LCM of a small number of reference samples may be ideal for isolating purified reference cell populations, and the resulting expression profiles can be used in conjunction with our computational approach for higher-throughput analysis of a large number of samples. While such an LCM-based method may provide improve estimates of compartment-based gene expression, however, the experimental validation of results obtained in the current study suggests that even non-ideal reference samples can substantially improve the detection of intrinsically regulated genes.

A second caveat that applies specifically to the initial estimates of compartment size is that differentiation of a given cell type may lead to what essentially constitutes a new cell type. Adequate modeling of such a cell type would be unlikely using the original reference populations. This problem may particularly complicate deconvolution efforts when a cellular process causes one cell type to express markers that are characteristic of another cell type from the reference set. This phenomenon may account for the substantial increase in the estimated abundance of fibroblasts observed in the mammary gland following Ras activation. Rather than an increase in *bona fide *fibroblasts, this may instead reflect the expression of mesenchymal markers in mammary epithelial cells due to an epithelial-mesenchymal transition, as suggested by the increase in *Snail *expression that we observed in response to Ras activation. The converse problem – multiple cell types (such as normal vs. neoplastic epithelial cells) appearing as a single type (epithelial) – should also be considered. However, our results suggest that this latter case is more likely to be interpretable using sensitive methods for detecting differential expression after correcting for other, more biologically distinct, cell populations.

## Conclusion

Our extension of the expression deconvolution approach, in conjunction with deconvolution-adjusted expression analysis, demonstrates the ability to correct microarray data obtained from a complex, heterogeneous mammalian organ for changes in compartment sizes. Beyond identifying changes in tissue compartments, this approach permits the improved detection of differentially expressed genes. Finally, these adjusted differential expression estimates identify statistical associations with functional annotation that suggest novel aspects of mammary gland biology and carcinogenesis. Our findings indicate that this model of expression deconvolution provides a powerful tool for the study of complex cellular mixtures in higher organisms.

## Methods

### Cell culture and tissue harvest

The non-transformed murine mammary epithelial cell (MEC) line, NMuMG [37], was cultured in Dulbecco's modified Eagle's medium (DMEM) supplemented with 10% bovine calf serum, 1% penicillin/streptomycin, and 2 mM L-Glutamine.

FVB/N (Taconic) mice were housed under barrier conditions with a 12 hr light/dark cycle and access to food and water ad libitum. Brown adipose tissue (BAT) was obtained from the interscapular fat pad of FVB mice. White adipose tissue (WAT) was obtained from sacral fat depots. Harvested tissues were snap frozen for RNA isolation.

Tissues used for microarray analyses of murine mammary gland development included independent, triplicate pools of mammary tissue from 10-week-old adult males as well as females at 12 time points of mammary gland development representing puberty, pregnancy, lactation, and postlactational involution [7]. All animal work described in this paper was carried out under humane conditions and has been approved by the University of Pennsylvania Laboratory Animal Resources committee.

Doxycycline-inducible systems that permit conditional gene expression in bitransgenic mice have been described [17]. The bitransgenic mouse line, MTB/TRAS, carries two transgenic constructs. The first, MMTV-rtTA (MTB), expresses the reverse tetracycline transactivator (rtTA) under the control of the murine mammary tumor virus (MMTV) promoter. The second, TetO-v-Ha-Ras (TRAS), expresses v-*Ha-Ras *under the control of the tetracycline-dependent minimal promoter. When MTB/TRAS mice are given 2 mg/ml doxcycline in drinking water, *Ras *is rapidly and specifically induced in the mammary gland [17]. The third, fourth, and fifth mammary glands were harvested from MTB/TRAS mice at days 0, 1, 2, 4, 8, and 14 of doxcycline treatment. At each time point, independent triplicate samples were prepared with each sample consisting of tissue pooled from 3 mice.

### Mammary whole mounts and immunostaining

Mammary glands were mounted on glass slides, fixed in 4% neutral buffered paraformaldehyde overnight, and transferred to 70% ethanol. Whole mounts were stained with carmine alum as described [9].

Mammary glands embedded in OCT were sectioned at 8 μm and fixed for 10 min in 4% neutral buffered paraformaldehyde. After rinsing 3 times in PBS (10 minutes/rinse), sections were treated in 0.5% Triton X-100 for 20 min. Sections were then rinsed 3 times in PBS and incubated in blocking buffer (PBS, 5% BSA, 0.3% Triton X-100, and 10% normal goat serum) for 1.5 hr at room temperature. Rat anti-mouse cytokeratin 8 (DSHB, the University of Iowa) was diluted in blocking buffer and incubated on sections overnight at 4°C. Sections were rinsed 3 times in PBS/0.3% Triton X-100 and stained with 1:1000 Alexa 567 conjugated goat anti-rat IgG (Molecular Probes) at room temperature for 2 hr. Stained sections were rinsed once in PBS/0.3% Triton X-100 and twice in PBS. Nuclei were counterstained with 1 μg/ml Hoechst 33258 dye, mounted in Fluoromount-G (SouthernBiotech) and visualized using a Leica DMRXE microscope. All images were captured using identical settings.

### Microarray analysis

Total RNA from cell lines and tissues was isolated and purified as described [38]. cDNA was generated and biotinylated cRNA was hybridized to Affymetrix MG-U74Av2 oligonucleotide microarrays as described [7]. Data files and published CEL files representing gene expression profiles for other cell types were listed in Additional File 1. For murine development, previously generated RNA pools [7] were re-analyzed on MG-U74Av2 arrays. All raw data were analyzed using Affymetrix GeneChip5.0 (MAS5) with default normalization to a target signal of 150.

### Identification of tissue-specific genes

To identify cell type-specific genes, the two-tailed Student *t*-test was first used to compare all possible sample pairs representing different tissue and cell types. Transcripts were selected that were consistently expressed at levels within a tissue at least 5.0-fold higher compared to any other tissue or cell type analyzed at a significance of p < 0.0005. Transcripts were then excluded that were considered "Absent" by MAS5 or that had an MAS5 signal value <500 in samples from the tissue within which they were most highly expressed. Transcripts on the resulting list were ranked based on signal-to-noise ratio using the Z-score transformed data for each sample [29] where higher values reflect consistently higher expression in the given group compared to expression in all other cell types. For genes measured by multiple probesets, the probeset with the highest MAS5 signal was retained if probe sets were adjacent in rank; in all other cases, the highest-ranking probe set was used.

To eliminate poorly discriminating genes, the ranked gene list for each tissue and cell type was further filtered using the SAS procedure PROC STEPDISC with the backward method and default significance level (Version 9.1 of SAS System for Windows). The most discriminating genes were thereby selected that minimized the ratio of within-group (consistently high or low expression with a group of tissue samples) sum of squares to total sum of squares for the model. Genes for each tissue type that passed the backward stepwise discriminant analysis were considered to represent the most specific genes for that tissue for subsequent analysis. A crude estimate of the efficiency of this step was obtained using the leave-one-out cross validation method in SAS PROC DISCRIM with the POOL and CROSSVALIDATE options and pooled covariance matrix when calculating the squared distances.

### Computational estimation of proportional contributions from cell types

Expression values for cell-type-specific genes were obtained from each reference sample and averaged across samples obtained from the same cell or tissue type. These values were taken as estimates of basal expression for further work. All subsequent calculations were performed after first normalizing the expression in each sample (or averaged sample group) across all probesets such that the mean = 0 and the standard deviation = 1. Estimation of the relative proportions of each cell type was then performed by obtaining solutions to linear equations of the form **Ax **= **y**, where **A **is an *m *× *n *matrix of expression values (*m *genes × *n *reference groups), **y **is a vector of *m *values in the test sample, and **x **is the vector of *n *values reflecting the estimated relative proportions of each cell type within the mixture. Solutions for **x **were estimated using simulated annealing as described by Lu *et al*. [6]

### Deconvolution-adjusted expression analysis

Given a sample for which an estimate of the relative proportions of composite cellular compartments has been obtained, gene expression was normalized by subtracting the expected contribution for each component. That is, the difference in expression that would have occurred in the absence of changes in compartment size was estimated using gadj=gorig−∑k=1nxkek
 MathType@MTEF@5@5@+=feaafiart1ev1aaatCvAUfKttLearuWrP9MDH5MBPbIqV92AaeXatLxBI9gBaebbnrfifHhDYfgasaacH8akY=wiFfYdH8Gipec8Eeeu0xXdbba9frFj0=OqFfea0dXdd9vqai=hGuQ8kuc9pgc9s8qqaq=dirpe0xb9q8qiLsFr0=vr0=vr0dc8meaabaqaciaacaGaaeqabaqabeGadaaakeaacqWGNbWzdaWgaaWcbaGaemyyaeMaemizaqMaemOAaOgabeaakiabg2da9iabdEgaNnaaBaaaleaacqWGVbWBcqWGYbGCcqWGPbqAcqWGNbWzaeqaaOGaeyOeI0YaaabCaeaacqWG4baEdaWgaaWcbaGaem4AaSgabeaakiabdwgaLnaaBaaaleaacqWGRbWAaeqaaaqaaiabdUgaRjabg2da9iabigdaXaqaaiabd6gaUbqdcqGHris5aaaa@4815@ where *g*_*adj *_is an adjusted expression value, *g*_*orig *_is the observed expression value in the test sample, and *x*_*k *_and *e*_*k *_refer to estimated proportions and expression values in each of *n *reference cellular compartments, respectively. To identify genes for which observed expression changes were not due to changes in the abundance of its expressing cell types, Significance Analysis of Microarrays (SAM) [11] was used to compare sets of *g*_*adj *_from replicate measurements under various experimental conditions. Comparisons were performed with a False Discovery Rate (FDR) cutoff of 3%. A Java application that calculates adjusted gene expression based on estimated proportions of an arbitrary number of cellular components is available for public download [39].

### Association with Gene Ontology (GO) annotation

Statistical associations between GO annotation and lists of differentially expressed genes were identified using EASE [16]. Multiple testing correction was performed using within-system bootstrapping, and a final cutoff of p < 0.05 was used to identify statistically significant associations.

## Authors' contributions

MW collected the public microarray data, conducted the computational analysis, performed the experimental validation, and drafted the manuscript. SRM participated in the analysis and interpretation of computational deconvolution results and prepared the manuscript. LAC conceived the study, participated in its design, analysis, and coordination, and prepared the manuscript. All authors read and approved the final manuscript.

## Supplementary Material

Additional File 1 Sources of Affymetrix MG-U74Av2 raw data used in the identification of tissue-specific genes Click here for file

Additional File 2 **Genes significantly regulated in mouse mammary gland at pregnancy compared to nulliparity**. An Excel file containing all genes on Affymetrix MG-U74Av2 arrays that were analyzed by comparing mammary tissues of d18 pregnant and 10 wk nulliparous mice. The file lists the change calls of the genes before ("Pre-adj") and after ("Post-adj") taking the estimated changes of cell compartment sizes into account. Click here for file

Additional File 3 **GO terms significantly associated with genes activated by pregnancy**. An Excel file containing the GO terms significantly associated with genes whose expression were considered to be significantly regulated, both before ("Pre-adj") and after ("Post-adj") taking estimated changes in cell compartment sizes into account in the mammary glands of d18 pregnant and 10 wk nulliparous mice. Click here for file

Additional File 4 **Genes regulated by oncogenic Ras activation**. An Excel file containing genes on Affymetrix MG-U74Av2 arrays that were analyzed by comparing mammary tissues at d4 and d0 following Ras activation. The file lists the change calls of genes before ("Pre-adj") and after ("Post-adj") taking the estimated changes of cell compartment sizes into account. Click here for file

Additional File 5 **GO terms significantly associated with genes activated by oncogenic Ras**. An Excel file containing all GO terms significantly associated with genes whose expressions were considered to be significantly regulated, both before ("Pre-adj") and after ("Post-adj") adjusting for estimated changes in cell compartment sizes following four days of Ras activation in the mammary gland. Click here for file

## Figures and Tables

**Table 1:** Identification of cell type-specific genes. Genes are ranked from highest to lowest signal/noise ratio. The expression profiles of genes in bold face are highly correlated (Pearson Correlation Coefficient > 0.5) with the average profile of the tissue-specific genes across mammary development stages.

**Tissue/Cell**	**Affy ID**	**Gene Symbol**	**Description**
**MEC**	**94270_at**	**Krt1-18**	**keratin complex 1, acidic, gene 18**
	**101009_at**	**Krt2-8**	**keratin complex 2, basic, gene 8**
	101574_f_at	Serpina1a	serine (or cysteine) proteinase inhibitor, clade A, member 1a
	**99649_at**	**Gclc**	**glutamate-cysteine ligase, catalytic subunit**
	101025_f_at	Sprr2a	small proline-rich protein 2A
	160099_at	Lgals4	lectin, galactose binding, soluble 4
	100329_at	Serpina1d	serine (or cysteine) proteinase inhibitor, clade A, member 1d
	**96596_at**	**Ndrg1**	**N-myc downstream regulated gene 1**
	92550_at	Krt1-19	keratin complex 1, acidic, gene 19
	**96735_at**	**Stard10**	**START domain containing 10**

**BAT**	**99507_at**	**Ucp1**	**uncoupling protein 1, mitochondrial**
	**161519_f_at**		**Mus musculus cDNA, 3' end/clone = 1700127J22/clone_end = 3'**
	**95695_at**	**Slc25a20**	**solute carrier family 25, member 20**
	102431_at	Mapt	microtubule-associated protein tau
	**98527_at**	**Dci**	**dodecenoyl-Coenzyme A delta isomerase**
	**100967_at**	**Slc27a2**	**solute carrier family 27 (fatty acid transporter), member 2**
	99994_at	Cidea	cell death-inducing DFFA-like effector A
	**96879_at**	**Ogdh**	**oxoglutarate dehydrogenase (lipoamide)**
	**102668_at**	**Ppara**	**peroxisome proliferator activated receptor alpha**
	**98984_f_at**	**Gpd2**	**glycerol phosphate dehydrogenase 2, mitochondrial**

**WAT**	**102366_at**	**Retn**	**resistin**
	99112_at	Slc25a10	solute carrier family 25, member 10
	**94309_g_at**	**Fbln1**	**fibulin 1**
	**100464_at**	**3110043O21Rik**	**RIKEN cDNA 3110043O21 gene**
	**102707_f_at**	**Serpina3c**	**serine (or cysteine) proteinase inhibitor, clade A, member 3C**
	**160989_r_at**	**B430320C24Rik**	**RIKEN cDNA B430320C24 gene**
	**99637_at**	**Col15a1**	**procollagen, type XV**
	**96742_at**	**Dpt**	**dermatopontin**
	**97835_at**	**Rarres2**	**retinoic acid receptor responder (tazarotene induced) 2**
	**96132_at**	**AB023957**	**cDNA sequence AB023957**

**3T3 Cells**	**101975_at**	**Dlk1**	**delta-like 1 homolog (Drosophila)**
	104286_at	Slc38a4	solute carrier family 38, member 4
	**104544_at**	**4930517K11Rik**	**RIKEN cDNA 4930517K11 gene**
	94338_g_at	Gas2	growth arrest specific 2
	**99051_at**	**S100a4**	**S100 calcium binding protein A4**
	**103327_at**	**Prrx2**	**paired related homeobox 2**
	**93574_at**	**Serpinf1**	**serine (or cysteine) proteinase inhibitor, clade F, member 1**
	**93681_at**	**Fzd2**	**frizzled homolog 2 (Drosophila)**
	**97943_at**	**Capn6**	**calpain 6**
	160939_at	Twist2	twist homolog 2 (Drosophila)
	160095_at	Lox	lysyl oxidase
	99435_at	Fgf7	fibroblast growth factor 7
	98501_at	Il1rl1	interleukin 1 receptor-like 1

**B Cells**	99945_at	Cd19	CD19 antigen
	**98035_g_at**	**H2-DMb1**	**histocompatibility 2, class II, locus Mb1**
	101878_at	Cd72	CD72 antigen
	**104429_at**	**Tap2**	**transporter 2, ATP-binding cassette, sub-family B (MDR/TAP)**
	**103040_at**	**Cd83**	**CD83 antigen**
	92741_g_at	Igh-6	immunoglobulin heavy chain 6 (heavy chain of IgM)
	**102939_s_at**	**Cd22**	**CD22 antigen**
	**92436_at**	**Stk23**	**serine/threonine kinase 23**
	**92359_at**	**Blk**	**B lymphoid kinase**

**Plasma cells**	**101656_f_at**	**Igk-V1**	**immunoglobulin kappa chain variable 1 (V1)**
	101870_at	Igh-4	immunoglobulin heavy chain 4 (serum IgG1)
	**92470_f_at**	**Igh-VJ558**	**immunoglobulin heavy chain (J558 family)**
	**102823_at**	**AU044919**	**M. musculus rearranged immunoglobulin gamma 2b heavy chain**
	**102154_f_at**	**Igk-V8**	**immunoglobulin kappa chain variable 8 (V8)**
	**97008_f_at**	**AI324046**	**expressed sequence AI324046**
	**100377_f_at**	**Igh-V**	**immunoglobulin heavy chain variable region**
	**96963_s_at**		**immunoglobulin light chain variable region**
	**97720_at**	**Igh-VS107**	**immunoglobulin heavy chain (S107 family)**
	**100299_f_at**	**LOC381783**	**Similar to immunoglobulin kappa chain**

**CD4+ Cells**	**100509_at**	**Rnf19**	**ring finger protein (C3HC4 type) 19**
	**93511_at**	**Itm2a**	**integral membrane protein 2A**
	**102661_at**	**Egr2**	**early growth response 2**
	101884_at	Xlr4	X-linked lymphocyte-regulated 4

**CD8+ Cells**	**162410_s_at**	**Cd8b**	**CD8 antigen, beta chain**
	98406_at	Ccl5	chemokine (C-C motif) ligand 5
	92398_at	BC026744	cDNA sequence BC026744
	99327_at	Prss19	protease, serine, 19 (neuropsin)
	**102975_at**	**Cd8a**	**CD8 antigen, alpha chain**
	**97995_at**	**Tcf7**	**transcription factor 7, T-cell specific**
	**92214_at**	**Ctsw**	**cathepsin W**
	**92406_at**	**Cd7**	**CD7 antigen**
	**103629_g_at**	**Lef1**	**lymphoid enhancer binding factor 1**
	**102904_at**	**H2-Ea**	**histocompatibility 2, class II antigen E alpha**

**Macrophage**	**103977_at**	**F10**	**coagulation factor X**
	101468_at	Pfc	properdin factor, complement
	92978_s_at	Serpinb2	serine (or cysteine) proteinase inhibitor, clade B, member 2
	95637_at	Flnb	filamin, beta
	93445_at	Cd5l	CD5 antigen-like
	**100397_at**	**Tyrobp**	**TYRO protein tyrosine kinase binding protein**
	**104541_at**	**Prtn3**	**proteinase 3**
	**103507_at**	**Emr1**	**EGF-like module containing, mucin-like, hormone receptor-like 1**
	95597_at	Ptgs1	prostaglandin-endoperoxide synthase 1
	**93097_at**	**Arg1**	**arginase 1, liver**
	**104354_at**	**Csf1r**	**colony stimulating factor 1 receptor**
	**96020_at**	**C1qb**	**complement component 1, q subcomponent, beta polypeptide**

**Table 2:** Alterations in change calls following adjustment for cell compartment size. Total probesets called upregulated (Up), downregulated (Down), or non-changing (NC) before ("Pre-adj") and after ("Post-adj") adjusting for estimated changes in cell compartment sizes. The percentages of probesets whose post-adjustment change calls were the same as or different than their pre-adjustment change calls are shown.

**Mammary glands**	**Pre-adj calls**	**Post-adj calls**	**Percentage of changed calls (%)**
d18 pregnant vs. 10 wk nulliparous	Up: 802	Up: 640 Down: 34 NC: 128	79.8 4.2 16.0
	Down: 1109	Up: 33 Down: 719 NC: 357	3.0 64.8 32.2
	NC: 10577	Up: 547 Down: 1085 NC: 8945	5.2 10.3 84.5

d4 vs. d0 following Ras activation	Up: 1545	Up: 1045 Down: 151 NC: 349	67.6 9.8 22.6
	Down: 792	Up: 85 Down: 330 NC: 377	10.7 41.7 47.6
	NC: 10151	Up: 970 Down: 947 NC: 8234	9.6 9.3 81.1

**Table 3:** Genes with altered change calls between d18 pregnant and 10 wk nulliparous glands after adjusting for compartment size changes.

**Affy ID**	**Symbol**	**Gene name**	**Pre-adj**	**Post-adj**
104587_at	Lama4	laminin, alpha 4	Down	Up
94354_at	Abca1	ATP-binding cassette, sub-family A (ABC1), member 1	Down	Up
93376_at	Bmp2k	BMP2 inducible kinase	Down	Up
93459_s_at	Fzd4	frizzled homolog 4 (Drosophila)	Down	Up
92913_at	Abcd2	ATP-binding cassette, sub-family D (ALD), member 2	Down	Up
98328_at	Tshr	thyroid stimulating hormone receptor	Down	Up
92836_at	Eln	elastin	Down	Up
97172_s_at	Abcc9	ATP-binding cassette, sub-family C (CFTR/MRP), member 9	Down	Up
98019_at	Tgfb1i1	transforming growth factor beta 1 induced transcript 1	Down	Up
100567_at	Fabp4	fatty acid binding protein 4, adipocyte	Down	NC
101887_at	Agt	angiotensinogen	Down	NC
94906_at	Adh1	alcohol dehydrogenase 1 (class I)	Down	NC
161900_f_at	Adrb3	adrenergic receptor, beta 3	Down	NC
94080_at	Sdha	succinate dehydrogenase complex, subunit A, flavoprotein (Fp)	Down	NC
98444_g_at	Lep	leptin	Down	NC
97426_at	Emp1	epithelial membrane protein 1	Down	NC
103549_at	Nes	nestin	Down	NC
92256_at	Ctsb	cathepsin B	Down	NC
97926_s_at	Pparg	peroxisome proliferator activated receptor gamma	Down	NC
97921_at	Agrn	agrin	Up	Down
100733_at	Psma2	proteasome (prosome, macropain) subunit, alpha type 2	Up	Down
95593_at	Golph2	golgi phosphoprotein 2	Up	Down
95286_at	Clu	clusterin	Up	Down
93789_s_at	Sin3b	transcriptional regulator, SIN3B (yeast)	Up	Down
99086_g_at	Usp3	ubiquitin specific protease 3	Up	Down
101137_at	Rps3	ribosomal protein S3	Up	Down
97937_at	Klf5	Kruppel-like factor 5	Up	Down
160129_at	Eef1d	eukaryotic translation elongation factor 1 delta	Up	Down
92544_f_at	Psma3	proteasome (prosome, macropain) subunit, alpha type 3	Up	NC
101446_at	Tpd52l1	tumor protein D52-like 1	Up	NC
94196_at	Ikbkg	inhibitor of kappaB kinase gamma	Up	NC
94404_at	Vps45	vacuolar protein sorting 45 (yeast)	Up	NC
103964_at	Esrra	estrogen related receptor, alpha	Up	NC
98937_at	Tbrg1	transforming growth factor beta regulated gene 1	Up	NC
102918_at	Muc1	mucin 1, transmembrane	Up	NC
94270_at	Krt1-18	keratin complex 1, acidic, gene 18	NC	Down
92550_at	Krt1-19	keratin complex 1, acidic, gene 19	NC	Down
101009_at	Krt2-8	keratin complex 2, basic, gene 8	NC	Down
98110_at	Mdm2	transformed mouse 3T3 cell double minute 2	NC	Down
93536_at	Bax	Bcl2-associated X protein	NC	Down
98433_at	Bid	BH3 interacting domain death agonist	NC	Down
99670_at	Bad	Bcl-associated death promoter	NC	Down
98868_at	Bcl2	B-cell leukemia/lymphoma 2	NC	Down
95611_at	Lpl	lipoprotein lipase	NC	Up
98623_g_at	Igf2	insulin-like growth factor 2	NC	Up
101874_s_at	Prlpe	prolactin-like protein E	NC	Up
104152_at	Stk40	serine/threonine kinase 40	NC	Up
97948_at	Rb1	retinoblastoma 1	NC	Up
99994_at	Cidea	cell death-inducing DNA fragmentation factor, alpha subunit-like effector A	NC	Up

**Table 4:** Biological Processes regulated in mammary glands of pregnant vs. nulliparous mice. Biological Process terms significantly associated with lists of genes that were detected as differentially expressed, either before ("Pre-adj") or after ("Post-adj") adjusting for estimated changes in cell compartment sizes in the mammary gland of d18 pregnant vs. 10 wk nulliparous mice.

**GO term**	**Pre-adj**	**Post-adj**
**(a) Upregulated genes**		
biosynthesis	X	
cellular biosynthesis	X	
establishment of localization		X
establishment of protein localization	X	X
intracellular protein transport	X	X
intracellular transport		X
localization		X
macromolecule biosynthesis	X	
protein biosynthesis	X	
protein localization	X	X
protein transport	X	X
translation	X	
transport		X
		
**(b) Downregulated genes**		
carboxylic acid metabolism	X	
cell adhesion	X	
cellular lipid metabolism	X	
defense response	X	
fatty acid metabolism	X	
generation of precursor metabolites and energy		X
immune response	X	
lipid metabolism	X	
organic acid metabolism	X	
response to biotic stimulus	X	

**Table 5:** Genes with altered change calls following four days of Ras activation after adjusting for compartment size changes.

**Affy ID**	**Symbol**	**Gene name**	**Pre-adj**	**Post-adj**
161497_f_at	Itga7	integrin alpha 7	Down	Up
99536_at	Cib2	calcium and integrin binding family member 2	Down	Up
103577_at	Pfkfb3	6-phosphofructo-2-kinase/fructose-2,6-biphosphatase 3	Down	Up
101015_s_at	Ifnar2	interferon (alpha and beta) receptor 2	Down	Up
98452_at	Flt1	FMS-like tyrosine kinase 1	Down	Up
93645_at	Rgs7	regulator of G protein signaling 7	Down	Up
99148_at	Fh1	fumarate hydratase 1	Down	Up
103556_at	Angptl2	angiopoietin-like 2	Down	Up
98418_at	Dvl1	dishevelled, dsh homolog 1 (Drosophila)	Down	NC
98447_at	Cebpa	CCAAT/enhancer binding protein (C/EBP), alpha	Down	NC
93392_at	Ucp3	uncoupling protein 3, mitochondrial	Down	NC
102016_at	Fsp27	fat specific gene 27	Down	NC
100567_at	Fabp4	fatty acid binding protein 4, adipocyte	Down	NC
98527_at	Dci	dodecenoyl-Coenzyme A delta isomerase	Down	NC
98444_g_at	Lep	leptin	Down	NC
97926_s_at	Pparg	peroxisome proliferator activated receptor gamma	Down	NC
101017_at	Cdk4	cyclin-dependent kinase 4	Up	Down
93536_at	Bax	Bcl2-associated X protein	Up	Down
100498_g_at	Stx3	syntaxin 3	Up	Down
102736_at	Ccl2	chemokine (C-C motif) ligand 2	Up	Down
98433_at	Bid	BH3 interacting domain death agonist	Up	Down
102976_at	Brca1	breast cancer 1	Up	NC
102911_at	Brca2	breast cancer 2	Up	NC
101009_at	Krt2-8	keratin complex 2, basic, gene 8	Up	NC
95412_at	Pdcd6	programmed cell death 6	Up	NC
104275_g_at	Trp53	transformation related protein 53	Up	NC
94448_at	Bcl10	B-cell leukemia/lymphoma 10	Up	NC
94270_at	Krt1-18	keratin complex 1, acidic, gene 18	Up	NC
99085_at	Usp3	ubiquitin specific protease 3	Up	NC
103238_at	Wnt4	wingless-related MMTV integration site 4	NC	Down
93515_at	Cdh16	cadherin 16	NC	Down
160430_at	Catnb	catenin beta	NC	Down
97766_at	Ros1	Ros1 proto-oncogene	NC	Down
102852_at	Cdh2	cadherin 2	NC	Down
92550_at	Krt1-19	keratin complex 1, acidic, gene 19	NC	Down
99670_at	Bad	Bcl-associated death promoter	NC	Down
92907_at	Ocln	occludin	NC	Down
101433_at	Mr1	major histocompatibility complex, class I-related	NC	Down
92752_r_at	Wnt10b	wingless related MMTV integration site 10b	NC	Down
94394_at	Rras	Harvey rat sarcoma oncogene, subgroup R	NC	Up
92256_at	Ctsb	cathepsin B	NC	Up
95663_at	Mmp2	matrix metalloproteinase 2	NC	Up
100320_at	Kpna4	karyopherin (importin) alpha 4	NC	Up
97765_g_at	Ros1	Ros1 proto-oncogene	NC	Up
162023_f_at	Thbd	thrombomodulin	NC	Up
104146_at	Rasip1	Ras interacting protein 1	NC	Up
98813_at	Rel	reticuloendotheliosis oncogene	NC	Up
99558_at	Ccnc	cyclin C	NC	Up
99467_at	Rasa1	RAS p21 protein activator 1	NC	Up

**Table 6:** Biological Processes regulated by Ras activation. Biological Process terms significantly associated with lists of genes that were detected as differentially expressed, both before ("Pre-adj") and after ("Post-adj") adjusting for estimated changes in cell compartment sizes in the mammary glands at d4 vs. d0 following Ras activation.

**GO Term**	**Pre-adj**	**Post-adj**
**(a) Upregulated genes**		
cell surface receptor linked signal transduction		X
cell-matrix adhesion		X
DNA metabolism	X	
inflammatory response		X
integrin-mediated signaling pathway		X
M phase of mitotic cell cycle	X	
mRNA processing	X	
protein amino acid phosphorylation		X
regulation of organismal physiological process		X
response to wounding		X
RNA processing	X	
signal transduction		X
		
**(b) Downregulated genes**		
antigen presentation, endogenous antigen	X	
ATP synthesis coupled electron transport	X	
carbohydrate metabolism	X	
cellular lipid metabolism	X	
cofactor metabolism	X	
electron transport	X	
fatty acid metabolism	X	
fatty acid oxidation	X	
glycolysis	X	
lipid metabolism	X	
nucleoside triphosphate metabolism		X
organic acid metabolism	X	
oxidative phosphorylation	X	
proton transport		X
purine nucleoside triphosphate metabolism		X
ribonucleoside triphosphate metabolism		X
sterol biosynthesis		X
tricarboxylic acid cycle	X	
