# Supplementary material for: Computational expression deconvolution in a complex mammalian organ
Source: BMC Bioinformatics. 2006 Jul 3;7:328. doi: 10.1186/1471-2105-7-328 (PMC1559723; doi:10.1186/1471-2105-7-328)
Supplement: Additional File 1 — Sources of Affymetrix MG-U74Av2 raw data used in the identification of tissue-specific genes [file 1471-2105-7-328-S1.doc]

Additional Table 1 − Sources of Affymetrix MG-U74Av2 raw data used in identification of tissue-specific genes

| Tissues/Cells | Number of Samples | CEL File Names in References | Reference |
| --- | --- | --- | --- |
| 3T3/Fibroblasts | 6 | 3T3 WT cont01, 3T3 WT cont02,  3T3 WT cont03, 3T3 WT cont04,  3T3 WT cont05, 3T3 WT cont06 | 1 |
| CD4+ T cells | 4 | GSM23439, GSM23440, GSM23441, GSM13442 | 2 |
| CD8+ T cells | 3 | CD8_Naive_01-44_1_A  CD8_Naive_01-44_1_B  CD8_Naive_01-44_1_C | 3 |
| B cells | 3 | LNB#1, LNB#2, LNB#3 | 4 |
| Plasma cells | 3 | PC#1, PC#2, PC#3 | 4 |
| Macrophage | 5 | PGA-MAA-IL4-1aAv2-s2  PGA-MAA-IL4-1bAv2-s2  PGA-MAA-IL4-1cAv2-s2  PGA-MAA-ctr-1bAv2-s2  PGA-MAA-ctr-1cAv2-s2 | 5 |
| Epithelial/NMuMG cells | 3 | N/A | 6 |
| BAT | 3 | N/A | 6 |
| WAT | 3 | N/A | 6 |

1. EBI ArrayExpress database (http://www.ebi.ac.uk/arrayexpress/). Accession number E-MEXP-135
2. Herman, AE, Freeman, GJ, Mathis, D, and Benoist, C. 2004. CD4+CD25+ T Regulatory Cells Dependent on ICOS Promote Regulation of Effector Cells in the Prediabetic Lesion. Journal of Experimental Medicine. 199: 1479-1489.
3. University of Virginia GEOSS public database (https://genes.med.virginia.edu/public_data/index.cgi).
4. Underhill GH, George, D, Bremer, EG, Kansas, GS. 2003. Gene expression profiling reveals a highly specialized genetic program of plasma cells. Blood. 101: 4013-4021.
5. Children’s National Medical Center-PGE database (<http://microarray.cnmcresearch.org/microarray.html>)
6. <http://www.afcri.upenn.edu/Chodosh/Docs/BMC_Bioinfo_2006/MAS5_MGU74Av2.txt>.
